# M2-like macrophages exert hepatoprotection in acute-on-chronic liver failure through inhibiting necroptosis-S100A9-necroinflammation axis

**DOI:** 10.1038/s41419-020-03378-w

**Published:** 2021-01-18

**Authors:** Li Bai, Ming Kong, Zhongping Duan, Shuang Liu, Sujun Zheng, Yu Chen

**Affiliations:** Beijing Municipal Key Laboratory of Liver Failure and Artificial Liver Treatment Research, the Fourth Department of hepatology, Beijing YouAn Hospital, Capital Medical University, Beijing, 100069 China

**Keywords:** Cell death and immune response, Liver fibrosis

## Abstract

Necroptosis has emerged as a novel and crucial player in acute and chronic liver diseases. Necroptotic cells lead to the release of DAMPs including S100A9, followed by the development of necroinflammation. We previously have documented the beneficial hepatoprotection conferred by M2-like macrophages in acute-on-chronic liver failure (ACLF) in vitro and in vivo, namely, M2-like macrophages protect hepatocytes against apoptosis. Herein, we integrated necroptosis, S100A9, and necroinflammation into this hepatoprotection, and hypothesized M2-like macrophages exert a hepatoprotective effect through inhibiting necroptosis-S100A9-necroinflammation axis. To testify this hypothesis, control mice were pre-treated with necroptosis or S100A9 inhibitors followed by D-GalN/LPS challenge. The extent of liver injury and M1/M2 macrophage activation was assessed. Necroptosis signaling and S100A9 expression were analysed and compared in control and fibrotic mice with or without acute insult. To document the pivotal role of M2-like macrophages in necroptosis and S100A9 inhibition, loss-of-function and gain-of-function experiments were performed. In addition, necroinflammation and its dependence on necroptosis and S100A9 were analysed. Moreover, the inhibitory effects of M2-like macrophages on necroinflammation were investigated in vivo and in vitro. We found that: firstly, the inhibition of necroptosis signaling and S100A9 expression alleviated D-GalN/LPS-induced hepatic damage, which was accompanied by M2-like macrophage activation; secondly, fibrosis inhibited necroptosis signaling and S100A9 expression, which could be attributed to M2-like macrophage activation; thirdly, S100A9 may function as a downstream player of necroptosis signaling; fourthly, fibrosis suppressed necroptosis- and S100A9-dependent necroinflammation; and finally, M2-like macrophages inhibited NLRP3 inflammasome activation and resultant necroinflammation via IL-10. Therefore, M2-like macrophages exert a beneficial hepatoprotection by inhibiting necroptosis-S100A9-necroinflammation axis in ACLF. Our findings provide novel insight for treating ACLF patients by specially targeting this signaling axis.

## Introduction

Acute-on-chronic liver failure (ACLF) refers to a severe acute deterioration of the established chronic liver disease, and usually develops after an acute insult^1,2^. So far, the pathogenesis of this severe disease remains to be elucidated. In the past few years, we have focused on elucidating this issue from the viewpoint of M1/M2 macrophage activation. Our major findings are as follows: firstly, hepatic fibrosis protects mice against diverse lethal insults; secondly, the hepatoprotection conferred by hepatic fibrosis can be attributed to M2-like activation of macrophages; and finally, M2-like macrophages derived from mouse livers and human PBMCs protect hepatocytes against apoptosis^3–5^.

Cell death is a hallmark pathological changes occurring in acute and chronic liver diseases^6,7^. Necrosis and apoptosis are two well-established forms of cell death. Most recently, several new modes of cell death, named regulated necrosis, have been discovered and characterized, including necroptosis, pyroptosis and ferroptosis^8–11^. Necroptosis has been shown to involve in embryonic development, tissue homeostasis, immunity, and inflammation^12,13^. The execution of necroptosis depends on the recruitment and deubiquitylation of the receptor-interacting serine/threomine kinase (RIPK)1. RIPK1 then autophosphorylates and recruits RIPK3 to develop necrosome. Upon phosphorylation by active RIPK3, the effector protein named mixed lineage kinase domain (MLKL) oligomerizes and translocates to cellular membranes, and finally results in cell rupture and necrosis^14–16^. Lately, an increasing number of studies explore the possible effects of this cell death mode in acute and chronic liver injury. Nevertheless, it remains conflicting and controversial whether necroptosis exists or not and its possible contribution to the pathogenesis of diverse liver diseases^14,17–20^.

The rupture of necroptotic liver cells is accompanied by the release of damage-associated molecular patterns (DAMPs). As a critical and novel DAMP molecule, S100-calcium-binding-proteinA9 (S100A9) has gained a lot of interest. S100A9 can signal tissue damage and trigger an innate immune response. The interaction between S100A9 and pattern recognition receptors Toll-like receptor 4 (TLR4) or receptor for advanced glycation endproducts (RAGE) promotes inflammatory and immunologic injury. Most recently, S100A9 has been identified as a critical player during multiple inflammatory liver diseases. Especially, serum S100A9 has been reported to be an interesting biomarker to monitor liver disease activity and to predict prognosis^21,22^.

NLR family pyrin domain containing 3 (NLRP3) represents the well-characterized inflammasome in the NLR family. Upon activation, NLRP3 oligomerizes and recruits apoptotic speck-like protein with a caspase-activating domain (ASC) into large oligomers called inflammasome complex. This complex can efficiently sense the intracellular signals such as DAMPs released by necroptotic cells, and engage the inflammatory caspase-1. Caspase-1 subsequently cleaves and activates the potent proinflammatory cytokines IL-1β and IL-18, thus induces their secretion and triggers profound necroinflammatory responses^23–26^.

We previously have documented that M2-like macrophages exert the beneficial hepatoprotective effects in ACLF, namely, M2-like macrophages protect hepatocytes against apoptosis. On the basis of the above-mentioned literature and our previous important findings, we integrated necroptosis, S100A9, and necroinflammation into the pathogenesis of ACLF, and hypothesized M2-like macrophages exert hepatoprotection in ACLF through inhibiting necroptosis-S100A9-necroinflammation axis. To verify this hypothesis, firstly, we documented the critical role of necroptosis and S100A9 in D-GalN/LPS-induced acute liver injury through the application of specific inhibitors; secondly, we analyzed the correlation between necroptosis or S100A9 inhibition and macrophage M1/M2 activation; thirdly, we demonstrated the inhibitory effects of fibrotic liver on necroptosis and S100A9 using mouse models of hepatic fibrosis and fibrosis regression; fourthly, the pivotal roles of M2-like macrophages in inhibiting necroptosis and S100A9 were verified through depleting and adoptively transferring M2-like macrophages; fifthly, the inhibitory effects of fibrosis on necroinflammation triggered by necroptosis and mediated by S100A9 were confirmed; finally, we dissected the role and possible mechanism by which M2-like macrophages suppressed the necroinflammation through in vivo and in vitro experiments. Our findings will shed new light on the pathogenesis of ACLF, and provide a novel therapeutic strategy for ACLF patients.

## Results

### Necroptosis inhibition alleviates acute liver injury, which is accompanied by M2-like activation of macrophages

**Fig. 1:**
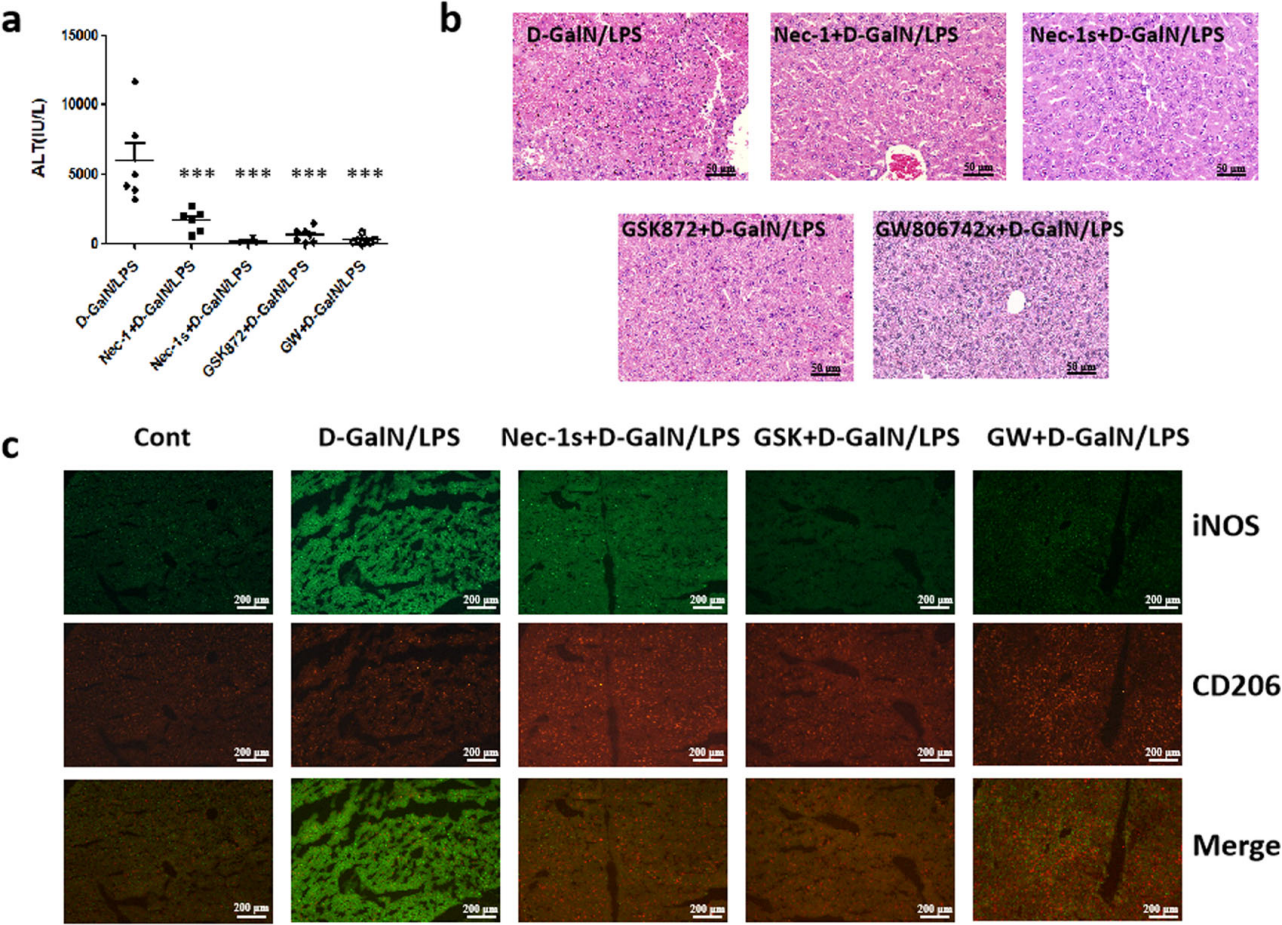
Necroptosis inhibition reduces acute hepatic injury triggered by D-GalN/LPS, which is closely related to M2-like activation of macrophages. **a** The hepatic damage assessed by serum ALT levels in mice challenged by D-GalN/LPS with or without necroptosis inhibitors. ***P < 0.001 compared with acutely injured mice. (*n* = 4–7) **b** The hepatic damage assessed by histopathology with H&E staining in mice challenged by D-GalN/LPS with or without necroptosis inhibitors. **c** The expression of M1 macrophage activation marker iNOS and M2 macrophage activation marker CD206 was detected by immunofluorescence in mice receiving D-GalN/LPS insult with or without necroptosis inhibitors.

First, we documented the existence of necroptosis in D-GalN/LPS-induced acute liver injury. Control mice were pre-treated with necroptosis inhibitors followed by D-GalN/LPS challenge. Compared with acutely injured mice, the protein and mRNA levels of RIPK3 and MLKL were significantly reduced in those mice pre-treated with necroptosis inhibitors (Supplementary Fig. 1a and 1b). Thus, D-GalN/LPS did trigger necroptosis, which could be inhibited successfully by specific inhibitors. We then assessed the effects of necroptosis inhibition on the extent of hepatic damage. Serum ALT and AST levels, which were remarkably elevated upon D-GalN/LPS challenge, were extremely suppressed in response to inhibitor pre-treatment (*P* < 0.001) (Fig. 1a and Supplementary Fig. 1c). In line with this, hepatic histology exhibited marked improvement in all groups receiving necroptosis inhibitors (Fig. 1b). Therefore, inhibiting necroptosis alleviates hepatic damage induced by D-GalN/LPS.

As key innate immune cells, macrophages assume essential roles in diverse liver diseases^27^. It is well-characterized that the M1/M2 balance of macrophage activation is a decisive factor for macrophage function^28^. Thus, we then analysed the activation phenotype of macrophages in mice receiving D-GalN/LPS challenge with or without necroptosis inhibitor. Stronger iNOS (M1 marker) and weaker CD206 (M2 marker) expression were noticed in the acutely injured liver. Diametrically, much weaker iNOS and stronger CD206 staining were observed in mice subjected to inhibitor pre-treatment (Fig. 1c). At the gene levels, M1 markers including IL-1β, TNF-ɑ, and IL-12 were obviously down-regulated in almost all groups receiving inhibitors compared to acutely injured mice; on the contrary, M2 markers including CD206, TGF-β, and YM-1 were up-regulated in mice receiving inhibitors, especially GW806742x pre-treatment (Supplementary Fig. 1d). Together, the inhibition of necroptosis is tied up with M2-like macrophage activation.

### Fibrosis suppresses necroptosis triggered by D-GalN/LPS, which can be attributed to M2-like macrophages

**Fig. 2:**
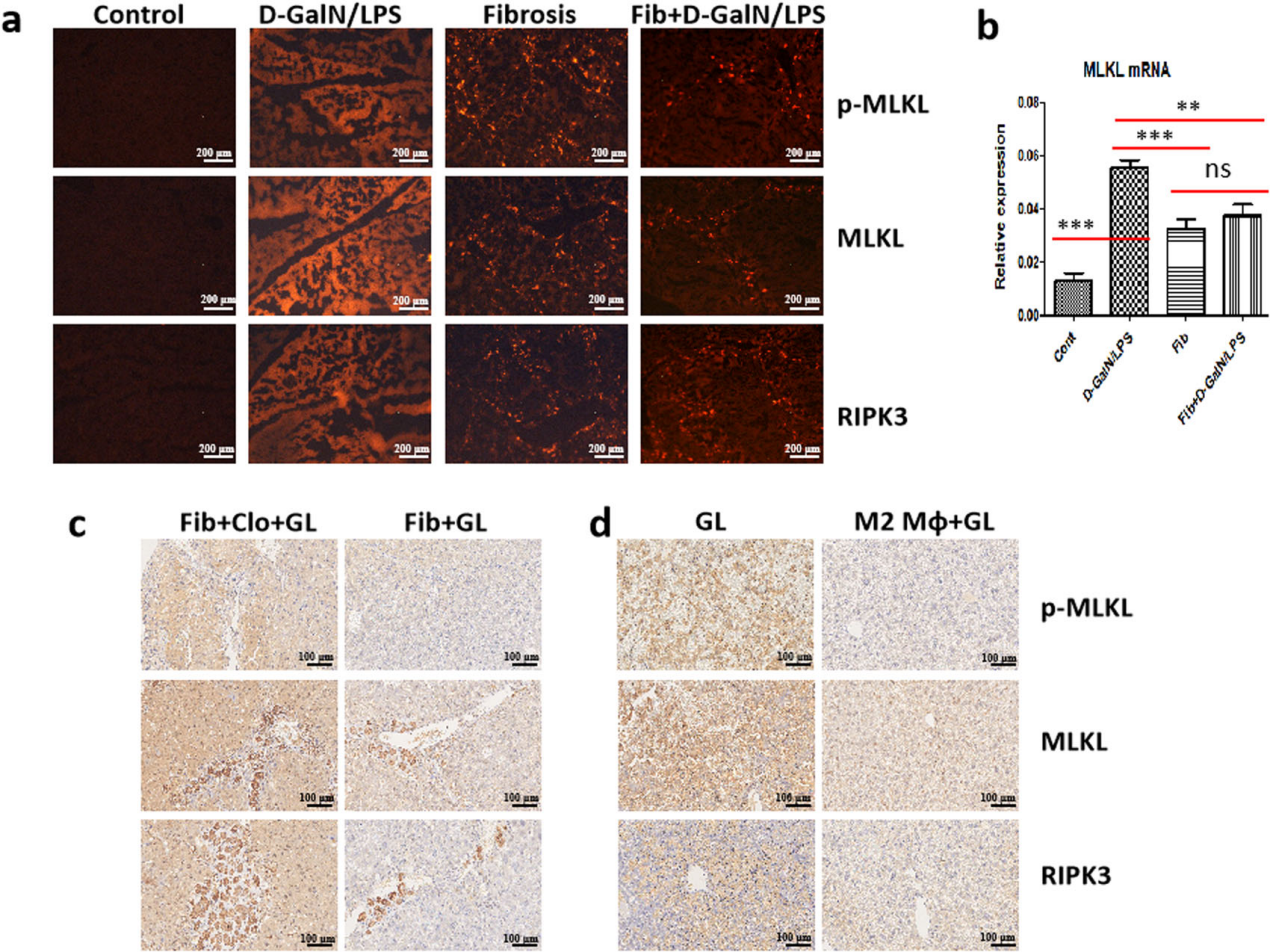
Fibrosis inhibits necroptosis signaling triggered by D-GalN/LPS, which can be attributed to M2-like macrophages. **a** The expression of necroptosis markers including RIPK3, MLKL, and P-MLKL in control and fibrotic mice with or without D-GalN/LPS challenge. **b** The mRNA levels of necroptosis marker MLKL in control and fibrotic mice with or without D-GalN/LPS challenge. (*n* = 5–7). **c** Comparison of the expression of necroptosis markers including RIPK3, MLKL, and P-MLKL in fibrotic mice upon acute insult with or without M2-like macrophage depletion (Clo: Liposome-encapsulated clodronate). **d** Comparison of the expression of necroptosis markers including RIPK3, MLKL, and P-MLKL in control mice upon acute insult with or without M2-like macrophage transfer. GL D-GalN/LPS.

We previously have demonstrated that hepatic fibrosis protects mice from diverse hepatotoxins^3^. Herein, we integrated necroptosis into this hepatoprotection, and intended to address the underlying mechanism governing this protective effect. Immunostaining showed that the protein expression of p-MLKL, MLKL, and RIPK3 was highly enhanced in mice upon D-GalN/LPS challenge, nevertheless, the expression of these signaling molecules was extremely attenuated in the fibrotic liver, even under acute challenge (Fig. 2a). Likewise, the mRNA levels of RIPK1, RIPK3, and MLKL were significantly inhibited in the livers of fibrotic mice (Fig. 2b and Supplementary Fig. 2a). On the other hand, hepatic expression of RIPK3, MLKL, and P-MLKL was faded in regressive mice, however, the levels of these markers enhanced gradually upon insult (Supplementary Fig. 2b). This association between weaker fibrosis and stronger necroptosis provided evidence for the pivotal role of liver fibrosis in necroptosis inhibition. Moreover, p-MLKL was found to be co-localised with SMA (Supplementary Fig. 2c), further supporting the correlation between necroptosis inhibition and hepatic fibrosis.

In our published work, we concluded that M2-like macrophages in the fibrotic liver confer mice great resistance to acute insults^3^. Herein, we wanted to confirm whether necroptosis inhibition conferred by hepatic fibrosis can also be attributed to M2-like macrophages. As a result, CD206 was co-localised with p-MLKL (Supplementary Fig. 2d), indicating M2-like macrophages may involve in the inhibition of necroptosis. Further, when we depleted macrophages in fibrotic mice (M2-like) followed by D-GalN/LPS challenge, the extent of necroptosis enhanced robustly as evidenced by much stronger expression of RIPK3, MLKL, and p-MLKL (Fig. 2c). On the other hand, when we adoptively transferred M2-like macrophages isolated from the fibrotic liver into normal mice followed by D-GalN/LPS injection, necroptosis was remarkably suppressed (Fig. 2d). Collectively, M2-like macrophages confer mice great resistance against necroptosis.

### The inhibition of S100A9, a downstream player of necroptosis signaling, alleviates acute liver injury, which is closely correlated with M2-like activation of macrophages

**Fig. 3:**
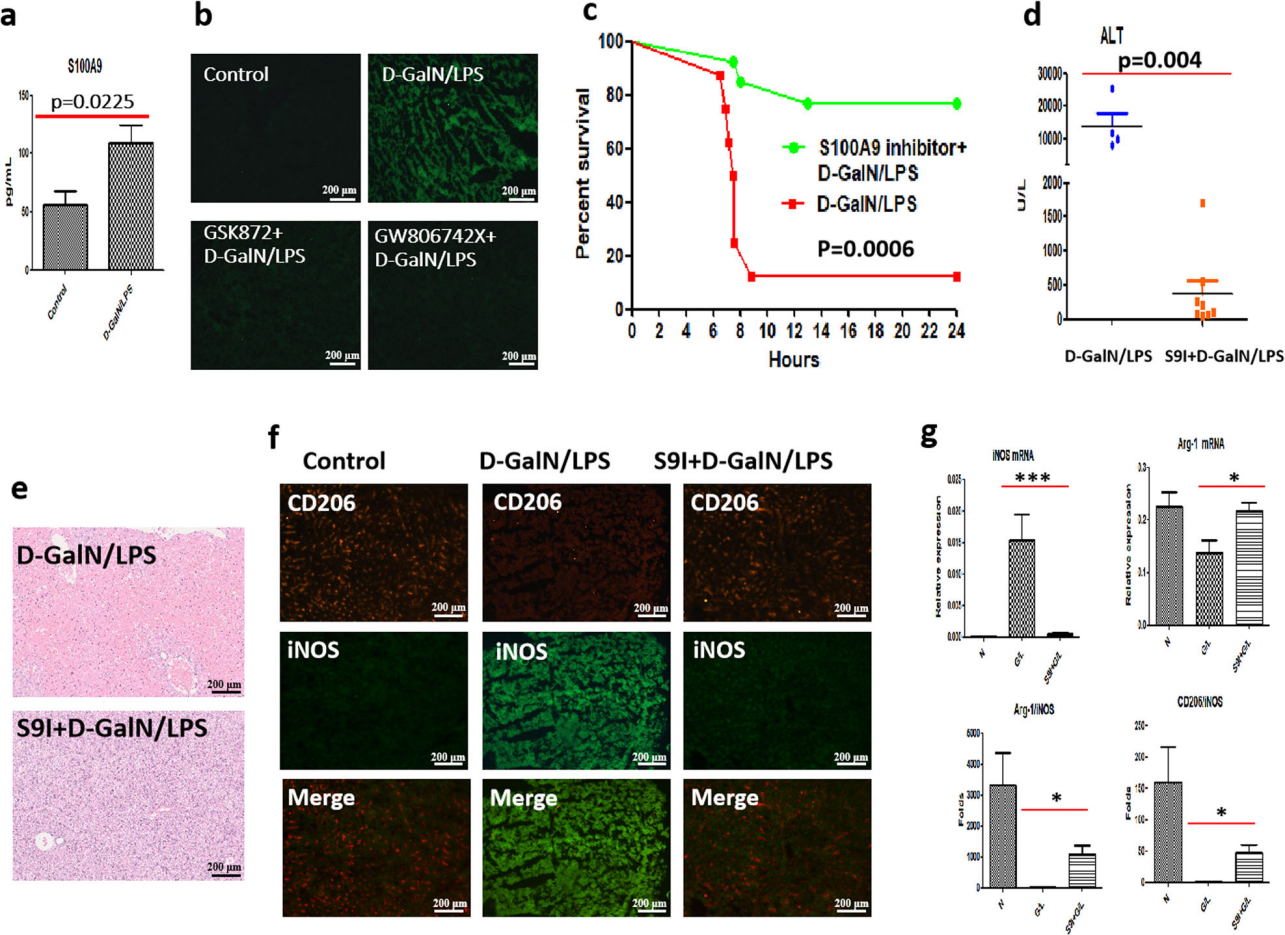
The inhibition of S100A9, a downstream player of necroptosis signaling, alleviates acute hepatic injury, which is relevant to M2-like activation of macrophages. **a** Serum S100A9 levels in control and acutely injured mice. (*n* = 6) **b** Comparison of S100A9 expression in the livers of acutely injured mice with or without necroptosis inhibitors. **c** Comparison of survival rates in acutely injured mice with or without S100A9 inhibitor. (*n* = 8–13) **d** The hepatic damage assessed by serum ALT levels in mice challenged by D-GalN/LPS with or without S100A9 inhibitors. (*n* = 5–8) **e** The hepatic damage assessed by histopathology with H&E staining in mice challenged by D-GalN/LPS with or without S100A9 inhibitors. **f** M1 macrophage activation marker iNOS and M2 macrophage activation marker CD206 expression in mice receiving D-GalN/LPS insult with or without S100A9 inhibitors. **g** M1 (iNOS) and M2 (Arg-1 and CD206) macrophage activation markers were assessed and compared by real-time PCR in mice receiving D-GalN/LPS insult with or without S100A9 inhibitors. *P < 0.05, ***P < 0.001. (*n* = 4–6).

The execution of necroptosis is accompanied by the rupture of the plasma membrane and the release of DAMPs. Next, we wanted to investigate whether S100A9 acts as a player in the hepatoprotection mediated by necroptosis inhibition. For this aim, we detected the level of S100A9 in D-GalN/LPS-induced acute liver injury. According to our data, serum S100A9 was remarkably up-regulated in acutely injured mice (Fig. 3a). Importantly, S100A9 expression was markedly reduced in mice pre-treated with RIPK3 inhibitor GSK872 or MLKL inhibitor GW806742X (Fig. 3b). This finding suggested S100A9 may function as a downstream molecule of necroptosis signaling.

In light of the aforementioned hepatoprotection mediated by necroptosis inhibition, we hypothesized S100A9 inhibition can alleviate hepatic damage triggered by D-GalN/LPS challenge. For this purpose, Paquinimod, an inhibitor specially targeting S100A9, was administrated intragastricly 2 h before and after D-GalN/LPS challenge. As a result, the expression and secretion of S100A9 were markedly reduced in mice receiving S100A9 inhibitor (Supplementary Fig. 3a and b). Thus, S100A9 expression can be inhibited effectively by the application of Paquinimod. Survival analysis showed that most of the mice (7 out of 8 mice, 87.5%) died upon D-GalN/LPS challenge, nevertheless, the mortality was only 23% (3 out of 13 mice) for mice receiving D-GalN/LPS plus Paquinimod (Fig. 3c, *P* = 0.0006). This finding provides powerful support for the critical role of S100A9 in acute liver injury. In accordance with this, serum ALT levels were obviously reduced in mice receiving Paquinimod treatment (Fig. 3d, *P* = 0.004). Moreover, liver histopathology was significantly improved in Paquinimod-treated mice (Fig. 3e). Therefore, S100A9 inhibition alleviates acute liver injury induced by D-GalN/LPS.

Considering the crucial role of M2-like macrophages in necroptosis inhibition, we next explored whether the alleviated hepatic damage mediated by S100A9 inhibition is also associated with M2-like activation of macrophages. Immunostaining showed that iNOS was obviously enhanced in the livers of acutely injured mice, meanwhile, the expression of CD206 was substantially diminished. Conversely, much weaker iNOS and stronger CD206 were observed in mice treated with Paquinimod (Fig. 3f). We also confirmed this finding at the transcriptional level. Obviously, downregulated M1 markers (iNOS and CD86) but markedly up-regulated M2 marker Arg-1 was detected in mice receiving S100A9 inhibitor. Specially, the ratio of M2/M1 markers (CD206/iNOS, Arg-1/iNOS, and YM-1/iNOS) tilted toward M2-like activation (Fig. 3g and Supplementary Fig. 3c). Therefore, S100A9 inhibition is closely tied up with M2-like macrophage activation.

### S100A9 expression is inhibited in the fibrotic liver, even under insult, which can also be ascribed to M2-like macrophage

**Fig. 4:**
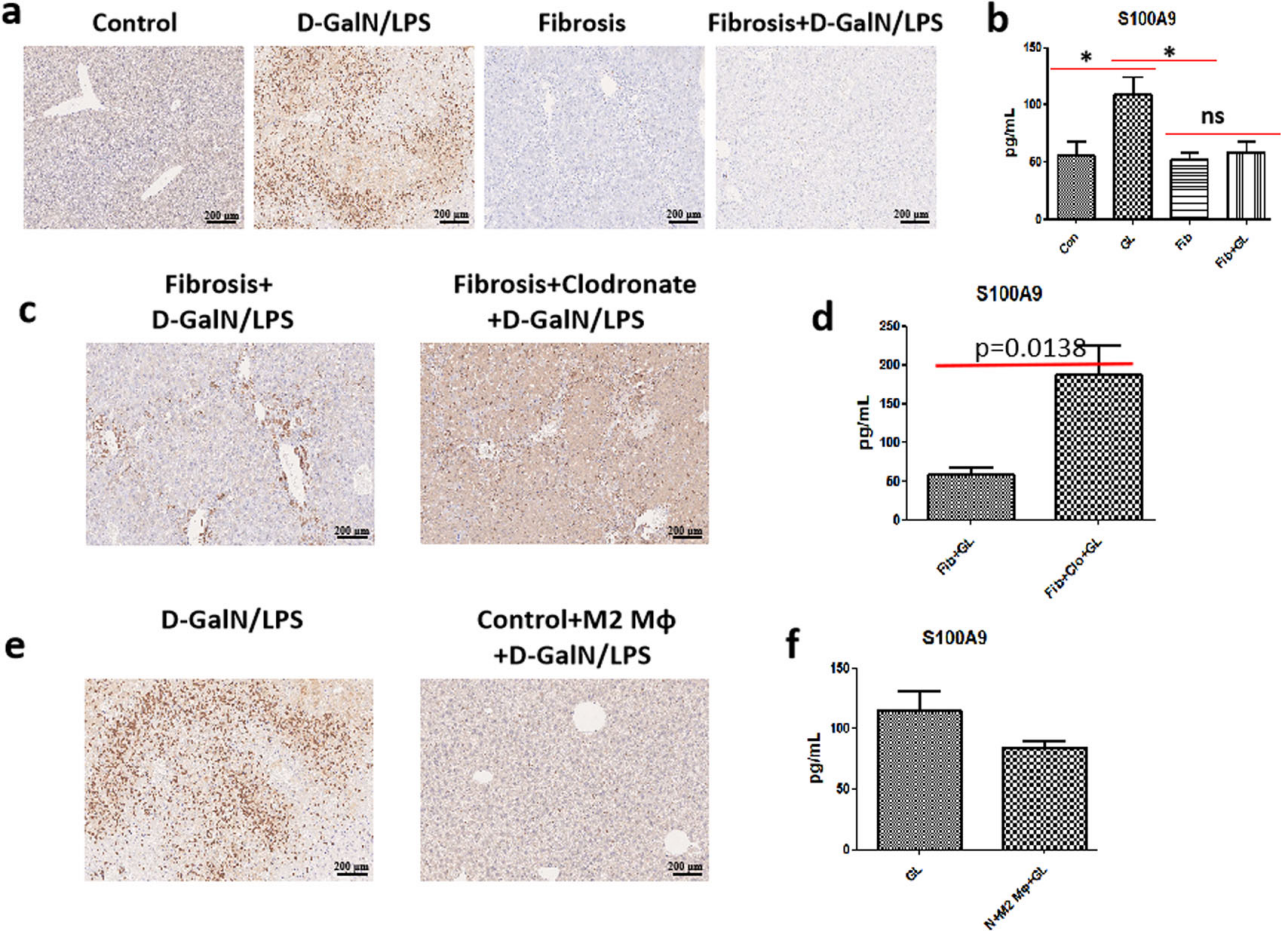
Fibrosis inhibits S100A9 expression, which can be ascribed to M2-like macrophages. **a**, **b** The expression of S100A9 in the livers of control and fibrotic mice with or without D-GalN/LPS challenge. (*n* = 4–7) **c**, **d** Comparison of S100A9 expression in fibrotic mice upon acute insult with or without M2-like macrophage depletion. (*n* = 4–7) **e**, **f** Comparison of S100A9 expression in control mice upon acute insult with or without M2-like macrophage transfer. (*n* = 4–7) GL D-GalN/LPS.

Similar to the inhibition of necroptosis signaling in fibrotic mice, S100A9 expression was probably suppressed in the fibrotic liver. As expected, S100A9 expression was sharply elevated in the acutely injured liver, nevertheless, it was remarkably inhibited in the fibrotic liver, even under acute challenge (Fig. 4a). Likewise, the secretion of S100A9 protein, which was significantly up-regulated in acutely injured mice, was obviously inhibited in fibrotic mice with or without acute insult (Fig. 4b). On the other hand, faded S100A9 expression in regressive mice was enhanced gradually upon insult (Supplementary Fig. 3d). Moreover, S100A9 expression was found to be co-localised with SMA in the fibrotic liver (Supplementary Fig. 3e). The above-mentioned evidence supports the close correlation between S100A9 inhibition and hepatic fibrosis.

We confirmed M2-like macrophages confer mice great resistance to necroptosis as stated earlier, herein, we attempted to verify whether S100A9 inhibition in the fibrotic liver can also be attributed to M2-like macrophages. As shown in Fig. 4c, the depletion of macrophages in fibrotic mice rendered the abolishment of S100A9 inhibition, as evidenced by the extremely enhanced expression of S100A9. In line with this, serum S100A9 levels were exceedingly elevated in fibrotic mice subjected to macrophage depletion (Fig. 4d). On the other hand, the adoptive transfer of M2-like macrophages resulted in the remarkable suppression of the expression and secretion of S100A9 (Fig. 4e, f). Collectively, S100A9 inhibition in fibrotic mice can also be ascribed to M2-like macrophages.

### M2-like macrophages in the fibrotic liver inhibit necroptosis-dependent and S100A9-mediated NLRP3 inflammasome activation and resultant necroinflammation

**Fig. 5:**
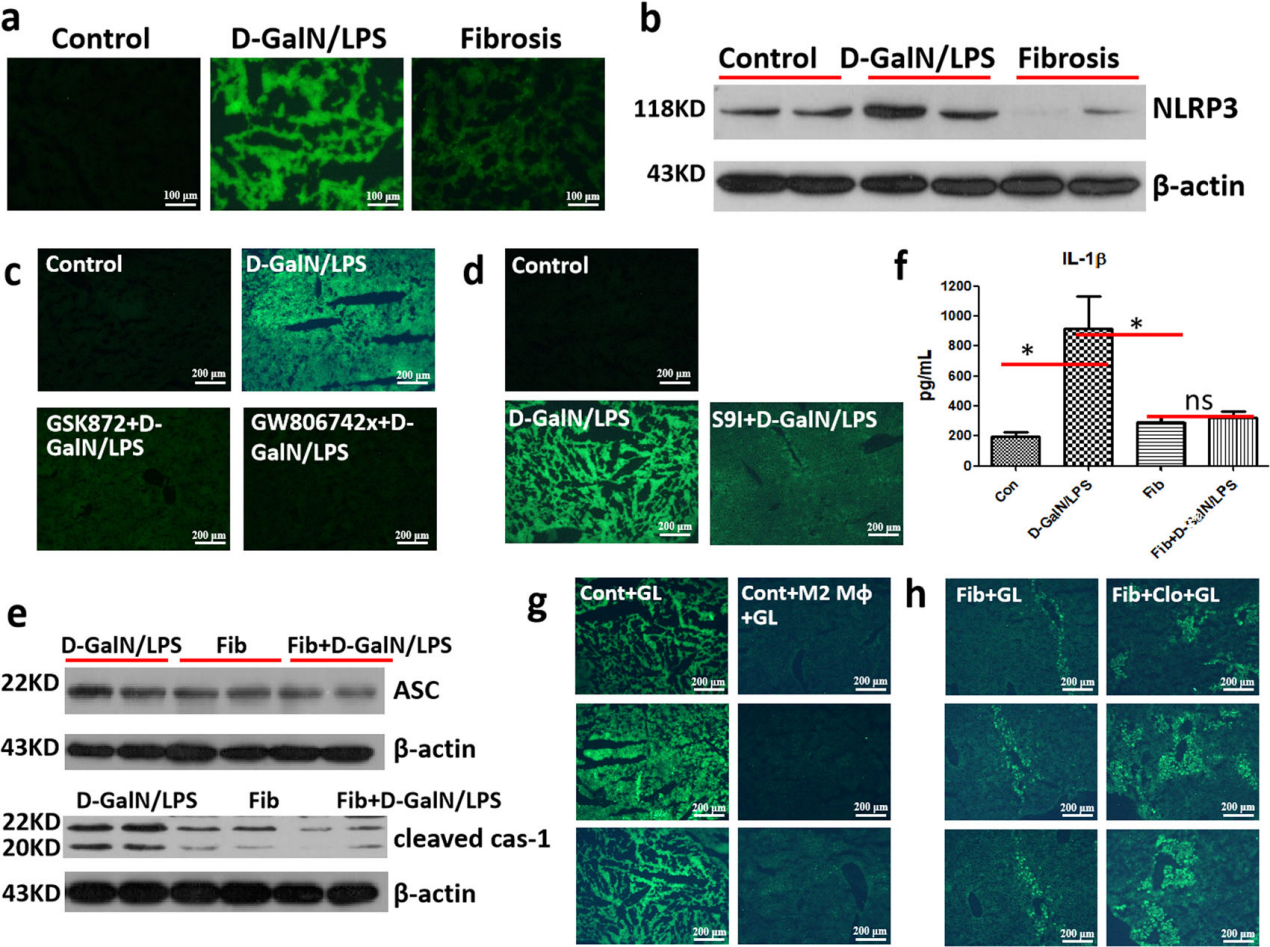
M2-like macrophages in the fibroic liver inhibit necroptosis- and S100A9-dependent NLRP3 inflammasome activation and ensuing necroinflammation. Comparison of NLRP3 expression in control, acutely injured, and fibrotic mice by immunofluorescence (**a**) and western blot (**b**) analysis. The expression of NLRP3 in mice receiving RIPK3 or MLKL inhibitor (**c**) and S100A9 inhibitor (**d**) followed by acute insult. **e** The expression of necroinflammation markers ASC and cleaved Caspase-1 in acutely injured mice and fibrotic mice with or without acute insult. **f** The expression of proinflammatory cytokine IL-1β in control and fibrotic mice with or without acute insult (*n* = 5–9). **g** Comparison of the expression of NLRP3 (upper), ASC (middle), and cleaved caspase-1 (lower) in acutely injured mice with or without M2-like macrophage transfer. **h** Comparison of the expression of NLRP3 (upper), ASC (middle), and cleaved caspase-1 (lower) in fibrotic mice upon acute insult with or without M2-like macrophage depletion.

RIPK3-MLKL signaling has been reported to promote inflammation by activating NLRP3 inflammasome^29,30^, however, it is not clear whether this signaling pathway plays a crucial role in our animal models. According to Fig. 5a, b, NLRP3, which was remarkably elevated in the acutely injured liver, was notably inhibited in the fibrotic liver, suggesting the pivotal role of hepatic fibrosis in suppressing NLRP3 inflammasome activation. Importantly, the administration of RIPK3 inhibitor GSK872 or MLKL inhibitor GW806742X brought about the significant suppression of NLRP3 expression in normal mice upon insult (Fig. 5c). In addition, pre-treatment with S100A9 inhibitor led to the obvious decreased expression of NLRP3 (Fig. 5d). Thus, NLRP3 activation was necroptosis-dependent and S100A9-mediated. When NLRP3 inflammasome activates, necroinflammation ensues^25^. Next, we assessed the level of necroinflammation in control and fibrotic mice with or without acute challenge. The protein levels of signal molecules related to necroinflammaton, especially cleaved caspase-1, were obviously reduced in the liver of fibrotic mice, even under insult (Fig. 5e). Similarly, obviously up-regulated IL-1β in the serum of acutely injured mice was significantly inhibited in the fibrotic mice with or without challenge (Fig. 5f). Together, fibrosis inhibits necroptosis-dependent and S100A9-mediated NLRP3 inflammasome activation and resultant necroinflammation.

Next, we dissected the potential mechanism by which fibrosis inhibits NLRP3 inflammasome activation and resultant necroinflammation. In light of the pivotal role of M2-like macrophages in necroptosis and S100A9 inhibition, we speculated that the inhibition of NLRP3 inflammasome activation and necroinflammation may also be ascribed to M2-like macrophages. To testify this speculation, we performed a depletion and adoptive transfer experiment of M2-like macrophages. Our data showed that the expression of NLRP3, ASC, and cleaved caspase-1 was significantly enhanced in fibrotic mice subjected to macrophage depletion and acute insult, compared to that in fibrotic mice upon insult (Fig. 5g); on the contrary, the levels of the above-mentioned indexes were sharply inhibited in M2-macrophages transferred mice compared to acutely injured mice (Fig. 5h). Thus, M2-like macrophages inhibit NLRP3 inflammasome activation and resultant necroinflammation.

### M2-like macrophages inhibit necroinflammation via IL-10 in vitro

**Fig. 6:**
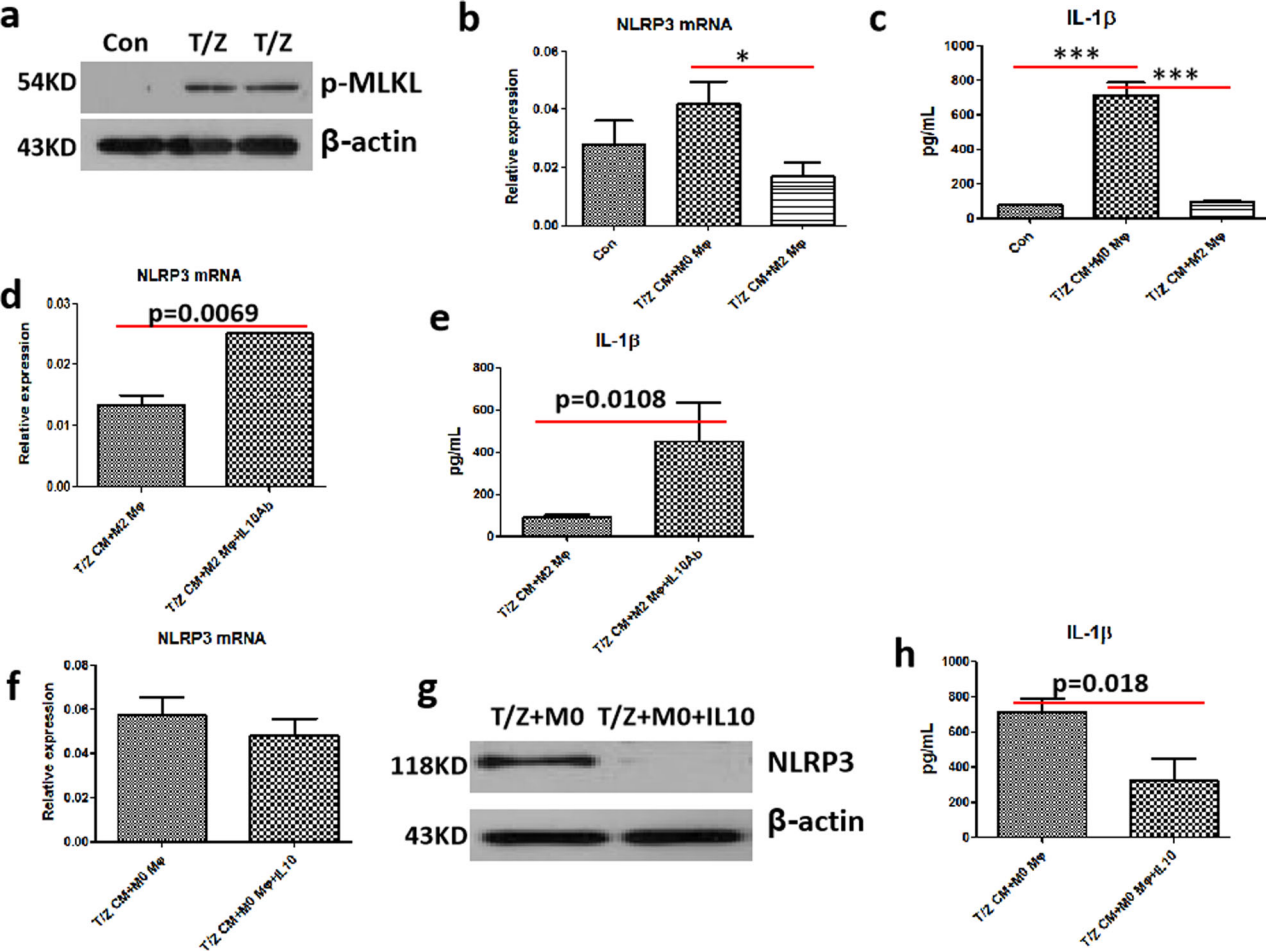
M2-like macrophages inhibit necroinflammation via IL-10 in vitro. **a** Necroptosis was induced by TNF-α/z-VAD (T/Z) in AML-12 hepatocytes, and the representative marker of necroptosis, p-MLKL, was detected by western blot analysis. **b**, **c** M0 or M2 macrophages were incubated with CMs from necroptotic AML-12 cells (T/Z CM), then the mRNA levels of NLRP3 and the protein levels of IL-1β were detected by real-time PCR (*n* = 3) and ELISA assay (*n* = 5–9), respectively. **d**, **e** M2 macrophages were incubated with CMs from necroptotic AML-12 cells (T/Z CM) and IL-10 antibody, the gene levels of NLRP3 (*n* = 4) and the protein levels of IL-1β (*n* = 4–9) were detected by real-time PCR and ELISA, respectively. **f**–**h** M0 macrophages were incubated with CMs from necroptotic AML-12 cells (T/Z CM) and recombinant mouse IL-10 cytokine, the gene and protein levels of NLRP3 were assessed by real-time PCR (*n* = 3) and western blot, respectively. The protein levels of IL-1β were assessed by ELISA (*n* = 4–8).

To further decipher the mechanism by which M2-like macrophages inhibit necroinflammation, we conducted in vitro conditioned medium (CM) experiments. Necroptosis of mouse hepatocytes was induced by TNF-α plus z-VAD (pan-caspase inhibitor) (Fig. 6a), then conditioned media from necroptotic hepatocytes were incubated with M0- or M2-polarized mouse macrophages. NLRP3 activation and ensuing necroinflammation in macrophages were assessed by real-time PCR and ELISA assay. As expected, NLRP3 mRNA and IL-1β levels were strikingly reduced in M2-like macrophages incubated with CM from necroptotic AML-12, compared with that in M0 macrophages (Fig. 6b, c). Nevertheless, when we neutralised IL-10 secreted during M2 macrophage activation with IL-10 antibody, the above-mentioned inhibition of NLRP3 and IL-1β was abolished (Fig. 6d, e). To further verify the substantial role of IL-10 in suppressing NLRP3-mediated necroinflammation, recombinant IL-10 was administrated into M0 macrophages incubated with CMs from necroptotic hepatocytes. As a result, the administration of IL-10 restored the inhibition of NLRP3 and IL-1β (Fig. 6f–h). Therefore, M2-like macrophages inhibit NLRP3 inflammasome activation and resultant necroinflammation through IL-10.

## Discussion

In the present study, we documented that M2-like macrophages exert hepatoprotection in ACLF through inhibiting necroptosis-S100A9-necroinflammation axis. To the best of our knowledge, this is the first time to integrate necroptosis-dependent and S100A9-mediated necroinflammation into M2-like macrophage-mediated hepatoprotection in ACLF.

Necroptosis has emerged as a novel and crucial player in liver diseases. Nevertheless, studies haven’t come to unanimous findings so far, and the precise role of necroptosis in liver injury remains controversial. For example, Ramachandran A and his colleagues reported that RIPK3 is a critical early mediator of acetaminophen-induced hepatocyte necrosis in mice^31^, however, Dara L et al. demonstrated that knockdown of RIPK3 and MLKL did not provide hepatoprotection as researchers expected^32^. These conflicting results motivate us to dissect the role and mechanism of necroptosis in our ACLF model. According to our data, necroptosis inhibition reduces hepatic damage induced by acute challenge, as evidenced by decreased serum ALT/AST levels and improved liver architecture. This finding strongly supports the vital role of necroptosis in D-GalN/LPS-induced liver injury.

Our published data have shown that macrophages assume the important but divergent functions in acute and chronic liver injury, which can be attributed to the diversity of macrophage phenotype^3^. Hence, we assessed and compared the activation phenotype of macrophages in mice upon insult with or without necroptosis inhibitors. All mice receiving inhibitor pre-treatment exhibit much weaker M1 but stronger M2 activation, which prompts the balance of macrophage activation to tilt toward M2-predominant phenotype. Thus, necroptosis inhibition is tied up with M2-like macrophage activation. Our finding is similar to the report from Yang et al.^33^, who show that defective necroptosis induces polarization bias in macrophages/microglia toward M2 phenotype in the ischemic cortex.

We have documented previously that fibrosis confers mice enhanced resistance to acute insults through inhibiting cell apoptosis^3^. In this study, we wanted to explore whether it also works when it comes to the correlation between hepatic fibrosis and necroptosis. Compared with acutely injured mice, much weaker expression of necroptosis signaling molecules is noticed in the fibrotic liver, even under acute insult. However, necroptosis inhibition is weakened significantly in regressive mice subjected to acute insult. Moreover, the co-localisation of SMA and P-MLKL provides further support for the interaction between fibrosis and necroptosis inhibition. Of note, necroptosis occurs in the setting of caspase 8 inhibition, which seems to argue the critical role of necroptosis in our ACLF model. Nevertheless, the latest report shows that apoptosis and necroptosis can coexist in APAP-induced liver injury depended on the different extent of hepatic damage^34^. Therefore, hepatic fibrosis does confer mice great resistance to necroptosis.

In view of the pivotal role of M2-like macrophages in hepatoprotection conferred by hepatic fibrosis, we then assessed the position of M2-like macrophages in necroptosis inhibition mediated by fibrosis. The co-localisation of CD206 and P-MLKL suggests the close association between M2-like macrophages and necroptosis. Specially, the data derived from loss-of-function and gain-of-function experiments (depletion and adoptive transfer of M2-like macrophages) provide reliable evidence for the crucial role of M2-like macrophages in necroptosis inhibition.

It has been well delineated that necroptosis is a type of lytic cell death. Thus, the execution of necroptosis is bound up with the rupture of the plasma membrane and the release of DAMPs^29,35^. In the present work, we investigated the potential role of DAMP in our model, and focused on S100A9 considering the reported importance of this DAMP molecule in multiple liver diseases^22,36,37^. According to our data, S100A9 holds a key place in D-GalN/LPS-induced acute liver injury, as evidenced by sharply decreased ALT levels and improved survival and histology in response to S100A9 inhibitor. S100A9 inhibition is accompanied by M2-skewed activation of macrophages, as exhibited by much enhanced expression of CD206 and higher M2/M1 ratio in the livers of mice receiving S100A9 inhibitor pre-treatment. Similar to necroptosis, S100A9 expression is obviously suppressed in the fibrotic mice with or without acute insult. And the gradual increase of S100A9 levels in regressive mice upon insult offers further support for the major contribution of hepatic fibrosis in S100A9 inhibition. Importantly, the loss-of-function and gain-of-function experiments provide powerful evidence for the decisive role of M2-like macrophages in S100A9 inhibition. Collectively, M2-like macrophages in the fibrotic liver confer mice beneficial hepatoprotection against acute insult through inhibiting S100A9.

In the present work, we demonstrated NLRP3 inflammasome is remarkably inhibited in the fibrotic liver, even under insult. Specially, we confirmed that the inhibition of NLRP3 inflammasome is necroptosis- and S100A9-dependent, which coincides with previous reports^29,30^. In agreement with this, necroinflammation is significantly suppressed in fibrotic mice with or without acute challenge, as shown by obviously reduced expression of ASC, cleaved caspase-1, and IL-1β. Therefore, hepatic fibrosis holds a pivotal place in suppressing necroptosis- and S100A9-dependent necroinflammation.

To elucidate the mechanism by which fibrosis confers hepatoprotection against necroinflammation, we performed the depletion and adoptive transfer experiments of M2-like macrophages. Enhanced expression of NLRP3, ASC, and cleaved caspase-1, which can reflect the severity of necroinflammation, was noticed in fibrotic mice subjected to M2-like macrophage depletion. Conversely, adoptive transfer of M2-like macrophages leads to the suppression of the above-mentioned indexes in normal mice upon insult. Hence, the inhibition of necroptosis-dependent and S100A9-mediated necroinflammation can be ascribed to M2-like macrophages.

Finally, we further dissected the mechanism by which M2-like macrophages exert hepatoprotection against necroinflammation. According to our data, M2-like macrophages are resistant to necroinflammation triggered by necroptotic hepatocytes. Nevertheless, this hepatoprotection can be abolished by the application of an IL-10 antibody. On the other hand, the necroinflammation occurred in M0 macrophages incubated with necroptotic CMs can be remarkably suppressed by recombinant mouse IL-10. Hence, M2-like macrophages inhibit necroinflammation via IL-10.

**Fig. 7:**
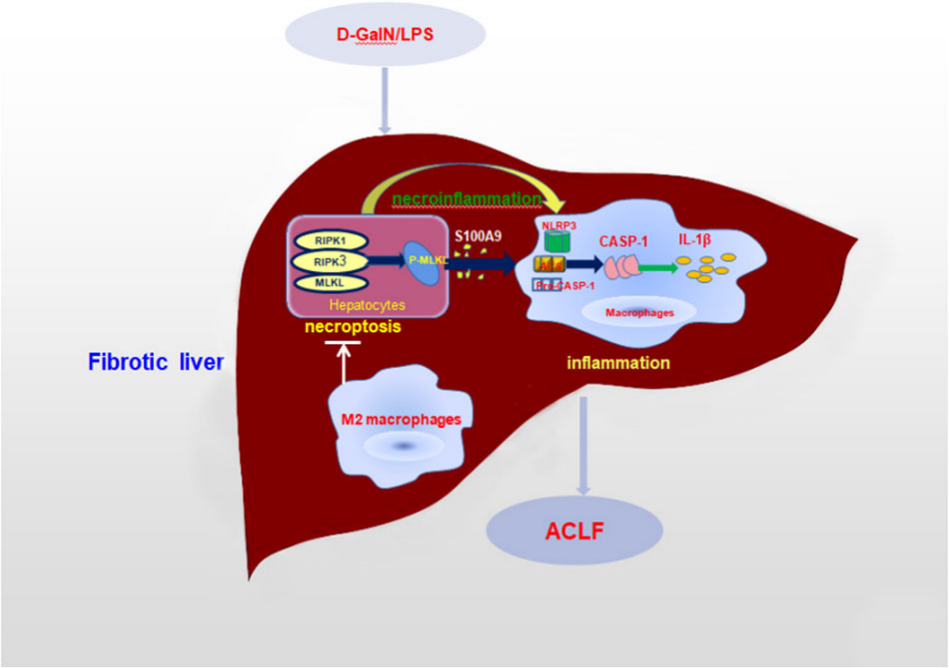
An illustration of the hepatoprotective role of M2-like macrophages through inhibiting necroptosis-S100A9-necroinflammation axis in ACLF. M2-like macrophages in the fibrotic mice inhibit necroptosis signaling and S100A9 expression triggered by acute insult, and further suppress necroptosis- and S100A9-dependent NLRP3 inflammasome activation and ensuing necroinflammation, therefore promoting injury resistance occurred in ACLF.

In brief, we conclude that M2-like macrophages in the fibrotic liver exert beneficial hepatoprotection in ACLF through inhibiting necroptosis-S100A9-necroinflammation axis (Fig. 7). Our findings offer novel opportunities for potential therapeutic intervention of ACLF by manipulating this signaling axis.

## Materials and methods

### Animals

BALB/c mice (male, 6–8-week old) were purchased from Laboratory Animal Center, Academy of Military Medical Sciences, Beijing, China. They were housed in a specific pathogen-free (SPF) environment under controlled conditions (22 °C–24 °C, 12-h-light/dark cycle). Animals were fed standard laboratory chow and water. All animal care and experimental procedures performed in this study were in accordance with the Guide for the Care and Use of Laboratory Animals, and approved by the Institutional Animal Care and Use Committee of Beijing YouAn Hospital, Capital Medical University.

### Animal treatment

Mice were treated with different reagents and divided into the following groups randomly: (1) BALB/c mice who received intraperitoneal injection of mineral oil, PBS or DMSO (as appropriate) were considered as a control group. (2) The blockade of necroptosis signaling: BALB/c mice were injected intraperitoneally with necroptosis inhibitors, namely, nec-1/nec-1s (5 mg/kg) targeting RIPK1, GSK872 (5 mg/kg) targeting RIPK3, and GW806742x (100 μM) targeting MLKL. (3) The inhibition of S100A9 expression: BALB/c mice were intragastricly administrated with Paquinimod, a special inhibitor for S100A9, 2 h before and after acute insult. (4) Induction of hepatic fibrosis: BALB/c mice were injected intraperitoneally with 20% CCl_4_ (2 μl/g, in mineral oil), twice a week, for 6 weeks. (5) Acute insult: Control and fibrotic mice were challenged intraperitoneally with D-GalN (500 μg/g, Sigma) plus LPS (10 ng/g, Invivogen). Sera and liver tissues were harvested 24 h after acute challenge for analysis.

### Evaluation of liver injury

Serum ALT and AST levels were measured using a multiparameteric analyzer (AU 5400, Olympus, Japan) according to an automated procedure.

Liver tissues were fixed in 10% neutral-buffered formalin and were embedded in paraffin, then the sectioned tissues (5 μm) were stained with hematoxylin-eosin (H&E) using a standard protocol. Histological pictures were captured and analysed under light microscopy.

### SYBR Green quantitative reverse-transcriptase polymerase chain reaction (qRT-PCR)

TRIzol reagent (Invitrogen, Carlsbad, CA, USA) was administrated to extract total RNA from frozen liver tissues and cells. One μg RNA was reverse-transcripted into cDNA using AMV retrotranscriptase system (TakaRa, Dalian, Liaoning, China). qPCR reactions in triplicate were run in ABI StepOne Plus System (Applied Biosystems, Foster City, CA, USA) using SYBR Green reaction mix (TakaRa). In a final reaction volume of 20 μl, the following were added: 1× SYBR Green (TakaRa, Dalian, Liaoning, China), cDNA, 0.5 mM each primer, and ROX. The reaction conditions were as follows: 95 °C (10 min) followed by 40 cycles of 95 °C (15 s) and 60 °C (1 min). The primers were designed by Primer version 3.0 and listed in Supplementary Table 1. Relative expression of gene transcripts was calculated and normalized to the expression of the reference gene GAPDH.

### Immunofluorescence (IF) analysis

Immunofluorescence was performed on frozen liver sections, as previously described^38^. Liver sections were stained with the following primary antibodies: FITC Mouse Anti-iNOS/NOS Type II (1:200; BD Transduction Laboratories™, San Jose, CA, USA), PE anti-mouse CD206 (MMR) (1:200; BioLegend Inc., San Diego, CA, USA), Anti-actin, α-Smooth Muscle antibody (1:300; Sigma, St Louis, MO, USA), Anti-mouse MLKL (1:400; Biorbyt, San Francisco, CA, USA), Anti-MLKL (phospho S345) antibody (1:400; Abcam), Anti-RIPK3 (1:400; Abcam), RIP(D94C12) XP Rabbit mAb (1:400; Cell Signaling Technology, MA, USA), anti-NLRP3 antibody (1:300; Abcam), anti-ASC antibody (1:200, Cell Signaling Technology), and anti-cleaved caspase-1 (1:200; Cell Signaling Technology). For indirect immunofluorescent staining, liver sections were incubated with the following secondary antibodies: PE-conjugated donkey anti-rabbit IgG for p-MLKL, MLKL, RIPK3, and SMA (1:500); Alexa Fluor 488-conjugated donkey anti-rabbit IgG for p-MLKL, MLKL, RIPK3, RIPK1, NLRP3, ASC, and cleaved caspase-1 (1:500). Nikon Inverted Fluorescence Microscope ECLIPSE Ti and NIS-Elements F 3.0 Software (Nikon Corporation, Tokyo, Japan) were applied for image capture.

### Immunohistochemistry (IHC) analysis

After deparaffinization and rehydration, liver sections were treated with 5% H_2_O_2_ for 15 min, followed by antigen retrieval for 3 min in EDTA buffer using a pressure cooker. The nonspecific proteins were blocked with goat serum for 20 min at 37 °C. Then liver sections were incubated with the following primary antibodies overnight at 4 °C: RIPK3 (1:2000), MLKL (1:100), P-MLKL (1:1000), S100A9 (1:50), and ASC (1:50). After washing three times with PBS, the sections were incubated with horseradish-peroxidase-conjugated goat anti-rabbit secondary antibody (Zhongshan Golden Bridge Biotechnology Co., Ltd., Beijing, China) for 20 min at 37 °C. After washing, the sections were incubated with diaminobenzidine as a chromogenic substrate and were counterstained with haematoxylin, dehydrated, and stabilized with a mounting medium. Pictures were obtained using an Olympus Bx51 microscope (Olympus America, Melville, NY, USA) and cellSens standard software (version 1.4.1).

### Western blot analysis

Frozen liver tissues or cells were treated with protein extraction reagent supplemented with Halt™ protease inhibitor cocktail (Thermo Scientific, IL, USA). Aliquots of lysates were subjected to 10% SDS-PAGE, then transferred onto polyvinylidene difluoride (PVDF) membrane (Thermo Scientific, USA). The membranes were blocked with 5% skim milk (BD Bioscience, USA) for 2 h at room temperature, and then probed with the following primary antibodies overnight at 4 °C: P-MLKL (1:10,000; Cell Signaling Technology), NLRP3 (1:4000; Abcam), ASC (1:4000; Cell Signaling Technology), cleaved caspase-1 (Asp297) (D57A2) rabbit mAb (1:2000; Cell Signaling Technology), and β-actin (1:5000; Immunoway). Next, the membranes were incubated with HRP-conjugated anti-rabbit IgG for 1 h at room temperature. Bands were visualized using Luminol ECL reagent (Thermo Scientific).

### Isolation and purification of primary mouse macrophages

Primary mouse macrophages were isolated from the livers of mice by pronase (Roche Diagnostics GmbH, Mannheim, Germany) and collagenase (Sigma-Aldrich, St. Louis, MO, USA) digestion followed by differential centrifugation using our previously reported method^3^. Briefly, in situ perfusion was applied through the portal vein and superior vena cava with DMEM/F12 (Gibco, Grand Island, NY, USA) containing 0.5% Pronase and 0.04% type IV collagenase. Then, the liver was harvested, excised and digested with DMEM/F12 containing 10 μg/ml DNase (Sigma). Digested livers were passed through a 70 μm cell strainer (BD Falcon). The filtrate was centrifuged, then the supernatant was further centrifuged for acquiring macrophages. The resultant pellets were re-suspended with DMEM (Hyclone, Logan, Utah, USA), and then overlaid onto Percoll (Sigma) gradient (40% to 70%), and centrifuged at 1100 × *g* for 20 min. Liver NPC suspension was further overlaid onto Percoll gradient (25% to 50%), and centrifuged at 1800 × *g* for 30 min. The interface was harvested and stained with biotin-conjugated anti-F4/80 (eBiosciences, San Diego, CA, USA). F4/80+ macrophages were further purified by streptavidin-conjugated magnetic beads (Miltenyi Biotec, Auburn, CA, USA) according to the manufacturer’s protocols.

### Macrophage polarization

Macrophages isolated from the liver of normal mice (M0 Macrophage) were activated with 50 ng/mL IL-4 (Novoprotein). The phenotype of activated macrophages was identified through qRT-PCR analysis for the gene signature of representative markers.

### Depletion of macrophages

We used Liposome-encapsulated clodronate (Clo) (FormuMax Scientific Inc., Palo Alto, USA) to deplete macrophages. A single dose of 0.2 ml Clo was injected into mice through the tail vein. The mice injected with the same dose of phosphate-buffered saline (PBS)-encapsulated liposomes (PBS-lip) (FormuMax Scientific Inc., Palo Alto, USA) were used as control. Forty-eight hours later, mice were challenged with D-GalN/LPS.

### Adoptive transfer of macrophages

For adoptive transfer of macrophages, macrophages (3 × 10^6^) isolated from the fibrotic liver (M2-like) were injected intravenously into normal mice by way of the tail vein.

### Enzyme-linked immunosorbent assay (ELISA)

S100A9 in serum and IL-1β in serum and conditioned medium were detected using mouse S100A9 ELISA kit (Abcam) and mouse IL-1 beta ELISA kit (RayBiotech), respectively, according to the manufacturers’ protocols.

### Conditioned medium (CM) experiments

CMs from necroptotic hepatocytes were collected and centrifuged to remove cell debris. Afterwards, CMs were incubated with M0 or M2-like macrophages for 24 h. The supernatants and cells were harvested for analysis.

### Statistical analysis

Results were expressed as mean ± standard error of the mean. Group comparisons were performed using Student’s *t* test, Mann–Whitney *U* test, or One-way ANOVA, as appropriate. The survival rates were calculated using the Kaplan-Meier method, and survival curves were compared using the log-rank test. Statistics and graphs were generated using Prism 5.0 software (GraphPad Software Inc., San Diego, CA, USA). *P* < 0.05 was considered statistically significant.

## Supplementary information

Supplementary information

Supplementary Figure 1

Supplementary Figure 2

Supplementary Figure 3
