# Supplementary material for: M2-like macrophages exert hepatoprotection in acute-on-chronic liver failure through inhibiting necroptosis-S100A9-necroinflammation axis
Source: Cell Death Dis. 2021 Jan 18;12(1):93. doi: 10.1038/s41419-020-03378-w (PMC7814003; doi:10.1038/s41419-020-03378-w)
Supplement: Supplementary file 1 — Supplementary information [file 41419_2020_3378_MOESM1_ESM.docx]

**M2-like macrophages exert hepatoprotection in acute-on-chronic liver failure through inhibiting necroptosis-S100A9-necroinflammation axis**

Li Bai^#^, Ming Kong^#^, Zhongping Duan, Shuang Liu, Sujun Zheng^*^, Yu Chen^*^

Beijing Municipal Key Laboratory of Liver Failure and Artificial Liver Treatment Research, the Fourth Department of hepatology, Beijing YouAn Hospital, Capital Medical University, Beijing, 100069, China

**Supplementary Figure Legends**

**Supplementary Figure 1. Necroptosis inhibition reduces acute hepatic injury triggered by D-GalN/LPS, which is closely related to M2-like activation of macrophages.** (a) The inhibitory effects of necroptosis inhibitors Nec-1s, GSK872, and GW806742x on RIPK1, RIPK3, and MLKL/P-MLKL signaling molecules. (b) Real-time PCR analysis for necroptosis markers RIPK3 and MLKL in mice receiving D-GalN/LPS or D-GalN/LPS plus necroptosis inhibitors. *P<0.05, **P<0.01. (n=4-6) (c) The hepatic damage assessed by serum AST levels in mice challenged by D-GalN/LPS with or without necroptosis inhibitors. ***P<0.001. (n=4-7) (d) M1 (IL-1β, IL-12, TNF-α) and M2 (TGF-β, CD206, YM-1) macrophage activation markers were assessed by real-time PCR in mice receiving D-GalN/LPS insult with or without necroptosis inhibitors. *P<0.05, **P<0.01. (n=5-7)

**Supplementary Figure 2. Necroptosis triggered by D-GalN/LPS is inhibited in the fibrotic liver, which can be attributed to M2-like macrophages.** (a) The mRNA levels of necroptosis markers RIPK1 and RIPK3 in control and fibrotic mice with or without D-GalN/LPS challenge. (n=5-7) (b) Comparison of MLKL expression in fibrotic mice during the resolution phase with or without D-GalN/LPS challenge. (R9d stands for the ninth day during the resolution phase; R15d stands for the fifteenth day during the resolution phase) (c) The co-localisation of P-MLKL and SMA in the liver of fibrotic mice. (d) The co-localisation of P-MLKL and CD206 in the liver of fibrotic mice.

**Supplementary Figure 3. S100A9 inhibition alleviates D-GalN/LPS-induced acute hepatic injury, which is closely correlated to M2-like activation of macrophages.** (a and b) The inhibitory effect of S100A9 inhibitor on S100A9 expression detected by IHC and ELISA. (n=4-7) (c) M1 (CD86) and M2 (YM-1) macrophage activation markers were assessed and compared by real-time PCR in mice receiving D-GalN/LPS insult with or without S100A9 inhibitors. (n=4-6) (d) Comparison of S100A9 expression in fibrotic mice during the resolution phase with or without D-GalN/LPS challenge. (R9d stands for the ninth day during the resolution phase; R15d stands for the fifteenth day during the resolution phase) (e) The co-localisation of S100A9 and SMA in the liver of fibrotic mice.

**Supplementary Table 1. The primers used in this work**

| **Gene** | **Sense** | **Anti-sense** |
| --- | --- | --- |
| GAPDH | 5’- aac ttt ggc att gtg gaa gg -3’ | 5’- aca cat tgg ggg tag gaa ca -3’ |
| RIPK1 | 5’-ctg ggc ttc act gag tct ca -3’ | 5’- tca agt ggt tca gca ggt ct -3’ |
| RIPK3 | 5’- gta ctt gga ccc aga gct gt -3’ | 5’- ctg tca cac act gtt tcc cg -3’ |
| MLKL | 5’- aag aag aac ctg ccc gat ga -3’ | 5’- ctg gct gac atc tga aac gg -3’ |
| IL-1β | 5’- gcc cat cct ctg tga ctc at -3’ | 5’- agg cca cag gta ttt tgt -3’ |
| IL-12 | 5'- cag ctt ctt cat cag gga cat -3' | 5'- ctt gag gga gaa gta gga atg -3' |
| TNF-α | 5'- gcc tct tct cat tcc tgc ttg t -3' | 5'- ttg aga tcc atg ccg ttg -3' |
| TGF-β | 5’- ttg ctt cag ctc cac aga ga - 3’ | 5’- tgg ttg tag agg gca agg ac -3’ |
| iNOS | 5'- ggt ctt tga aat ccc tcc tga -3' | 5'- agc tcc tgg aac cac tcg ta -3' |
| Arg-1 | 5’- ctg gca gtt gga agc atc tct -3’ | 5’- gtg agc atc cac cca aat gac -3’ |
| CD206 | 5’- atg cca agt ggg aaa atc tg -3’ | 5’- tgt agc agt ggc ctg cat ag -3’ |
| YM-1 | 5’- atc tat gcc ttt gct gga atg c -3’ | 5’- tga atg aat atc tga cgg ttc tga g -3’ |
| NLRP3 | 5’- atg ctg ctt cga cat ctc ct -3’ | 5’- aac caa tgc gag atc ctg ac -3’ |
| CD86 | 5’- cac gag ctt tga cag gaa ca -3’ | 5’- tta ggt ttc ggg tga cct tg -3’ |
