# Supplementary figures and images for: M2-like macrophages exert hepatoprotection in acute-on-chronic liver failure through inhibiting necroptosis-S100A9-necroinflammation axis
Source: Cell Death Dis. 2021 Jan 18;12(1):93. doi: 10.1038/s41419-020-03378-w (PMC7814003; doi:10.1038/s41419-020-03378-w)

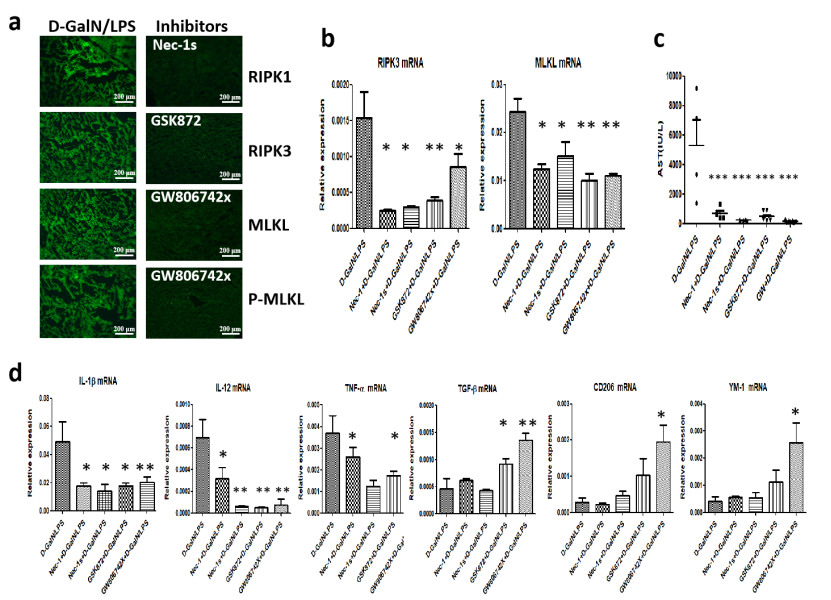

Supplement: Supplementary file 2 — Supplementary Figure 1 [file 41419_2020_3378_MOESM2_ESM.tif]

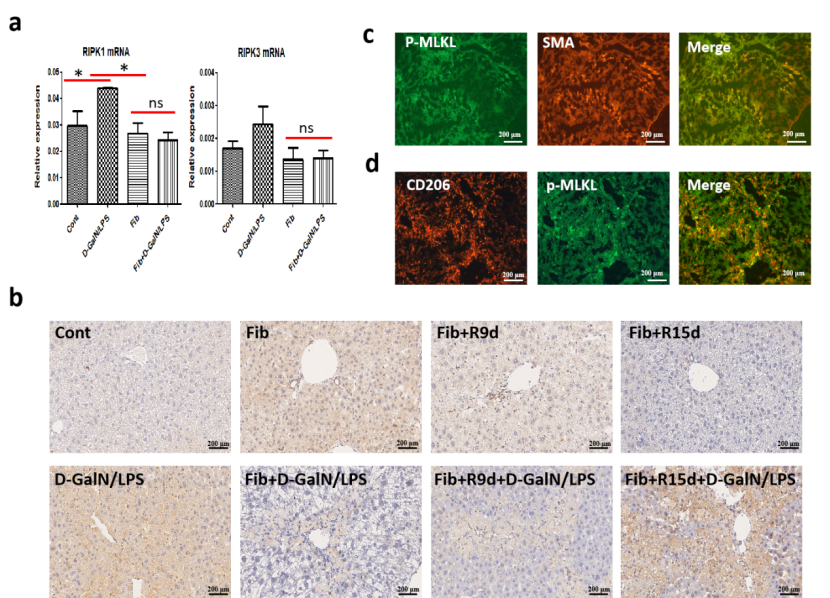

Supplement: Supplementary file 3 — Supplementary Figure 2 [file 41419_2020_3378_MOESM3_ESM.tif]

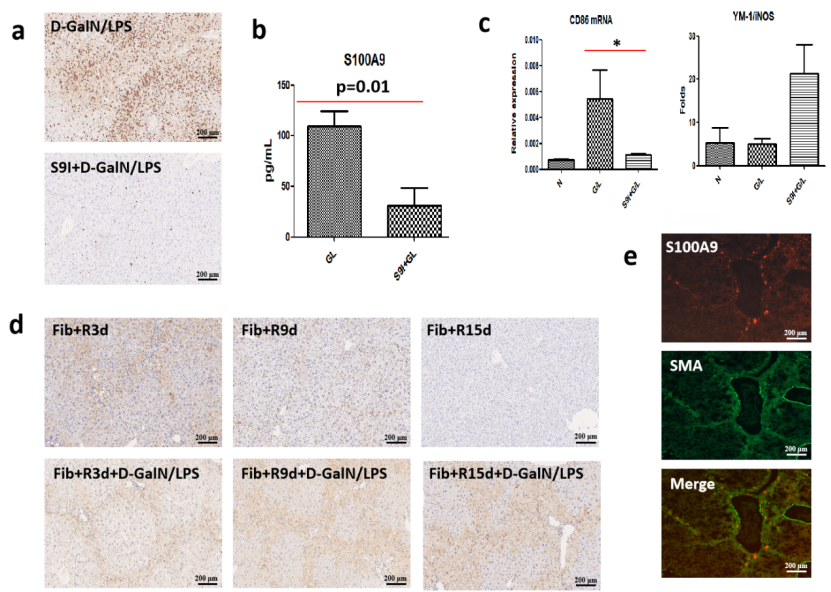

Supplement: Supplementary file 4 — Supplementary Figure 3 [file 41419_2020_3378_MOESM4_ESM.tif]
